# Estradiol and Bisphenol A Stimulate Androgen Receptor and Estrogen Receptor Gene Expression in Fetal Mouse Prostate Mesenchyme Cells

**DOI:** 10.1289/ehp.9804

**Published:** 2007-02-27

**Authors:** Catherine A. Richter, Julia A. Taylor, Rachel L. Ruhlen, Wade V. Welshons, Frederick S. vom Saal

**Affiliations:** 1 Division of Biological Sciences and; 2 Veterinary Biomedical Sciences, University of Missouri-Columbia, Columbia, Missouri, USA

**Keywords:** 17β-estradiol, androgen receptor gene, bisphenol A, dose–response relationship, estrogen receptor 1 (α) gene, prostate, sexual differentiation

## Abstract

**Background:**

Hormonal alterations during development have lifelong effects on the prostate gland. Endogenous estrogens, including 17β-estradiol (E_2_), and synthetic estrogenic endocrine disruptors, such as bisphenol A (BPA), have similar effects on prostate development. Increasing exposure to estrogens within the low-dose, physiologic range results in permanent increases in the size and androgen responsiveness of the prostate, whereas exposure within the high-dose, pharmacologic range has the opposite effects.

**Objectives:**

We tested the hypothesis that the low-dose effects of estrogens on the developing prostate are associated with increased expression of androgen receptor (*Ar*) and estrogen receptor 1 (α) (*Esr1*) genes in mesenchyme cells.

**Methods:**

Ar and Esr1 mRNA levels were quantified in primary cultures of fetal mouse prostate mesenchyme cells treated with E_2_ and BPA.

**Discussion:**

Ar and Esr1 mRNA expression increased in response to E_2_, with thresholds of 0.001 and 0.037 nM, respectively; and in response to BPA, with a threshold of 1 nM for both mRNAs. We did not observe the expected inhibition of Ar mRNA expression by pharmacologic levels of E_2_ relative to unexposed cells.

**Conclusions:**

The observed induction of gene expression occurred at concentrations within the range of free E_2_ previously shown to permanently increase prostate size, thus supporting the involvement of direct effects of estrogens on gene expression in prostate mesenchyme. The effects of BPA occurred within the range of concentrations currently measured in human serum, demonstrating the vulnerability of developing tissues to xenoestrogens.

During fetal life, alterations in normal prostate gland development can produce permanent changes that persist throughout adulthood and may increase the risk of disease in later life (Ho et al. 2006; Risbridger et al. 2005). The prostate differentiates from the cranial region of the urogenital sinus (UGS) (Marker et al. 2003). In humans, the first epithelial buds are observed in the lateral region of the UGS during the tenth week of gestation in a pattern that shows a remarkable similarity to that of bud development in mice and rats during the early phase of gland genesis (Timms et al. 1994). Prostate ductal budding begins on gestation day (GD) 17 in mice (2 days before birth) (Timms et al. 1994). Prostate development is dependent on 5α-dihydrotestosterone (DHT) production from testosterone within the UGS mesenchyme (Marker et al. 2003). Androgen receptor expression in prostatic mesenchyme is required for directing growth and branching morphogenesis of epithelial buds, presumably by induction of paracrine factors secreted by mesenchyme (Cunha and Donjacour 1987; Kokontis and Liao 1999). During development, prostatic epithelial cells exhibit little androgen binding, and androgen receptor protein expression in epithelium is not required for differentiation (Cunha and Donjacour 1987; Prins and Birch 1995; Timms et al. 1999). Therefore, fetal mouse UGS mesenchyme cells provide an informative model of endocrine control of prostate development.

There is now considerable evidence that estrogens modulate the activity of androgens in regulating prostate development. The UGS mesenchyme in mice and rats responds to estrogens via estrogen receptor 1 (α ), whereas in the human prostate estrogen receptor 2 (β ) may mediate most responses to estrogens during development (Adams et al. 2002; Prins et al. 1998). Prostatic growth and androgen receptor ligand-binding activity are permanently decreased in response to high, pharmacologic doses of both natural and xenobiotic estrogens during development (Prins and Birch 1995; Rajfer and Coffey 1978; vom Saal et al. 1997). In contrast, increases in prenatal estrogen levels within the physiologic range (the normal range for endogenous estradiol) stimulate prostate development, leading to permanently increased prostate size and androgen receptor ligand-binding activity (Gupta 2000; Timms et al. 1999; vom Saal et al. 1997).

Estrogenic endocrine disruptors have the potential to alter prostate development in a manner similar to that of endogenous estradiol. In this study, we chose to examine bisphenol A (BPA), the monomer used to make polycarbonate plastic and as an additive in many other plastic products. BPA is produced in excess of 6 billion pounds per year, and the potential for human exposure is great due to leaching from plastic and plastic-lined metal food and beverage containers, as well as from dental sealants (Takao et al. 2002; Welshons et al. 2006).

We have proposed that one mechanism by which fetal estrogen exposure stimulates prostate development is by increasing prostatic androgen receptor gene [*Ar*; GenBank accession no. X53779 (Benson et al. 2007)] expression, thereby increasing the androgen responsiveness of the developing prostate, leading to enhanced gland genesis and growth (Richter et al. 2005; vom Saal et al. 1997). In the present study we sought to determine whether the endogenous hormone 17β-estradiol (E_2_), within its physiologic range, and the manmade estrogenic endocrine disruptor BPA, within the range measured in human serum (Schönfelder et al. 2002), directly influence *Ar* and estrogen receptor 1 (α ) (*Esr1*; GenBank accession no. NM_007956.2) gene expression at the transcriptional level in fetal mouse UGS mesenchyme.

## Materials and Methods

### Animals, housing, mating, and organ collection

CD-1 mice were purchased from Charles River Laboratories (Wilmington, MA) and bred at the University of Missouri in a facility accredited by the Association for Assessment and Accreditation of Laboratory Animal Care. Animals were housed on corn-cob bedding in standard polypropylene cages. They received water purified by ion exchange and carbon filtration from glass bottles. Pregnant and lactating females were fed Purina 5008 chow (Purina Mills, St. Louis, MO). After being weaned, animals were fed Purina 5001 chow (Ralston Purina). Rooms were maintained at 25 ± 2°C under a 12 hr:12 hr light:dark cycle. Animals were treated humanely and with regard for alleviation of suffering. Animal procedures were approved by the University of Missouri Animal Care and Use Committee and conformed to the NIH *Guide for the Care and Use of Laboratory Animals* (Institute of Laboratory Animal Resources 1996).

### Tissue collection, primary cell culture, and dosing

Timed-pregnant females were killed on GD17 (mating = GD0) by CO_2_ asphyxiation, and fetuses were removed from the uterine horns. The bladder and UGS were removed from male fetuses as previously described (Timms et al. 1999; vom Saal et al. 1997). The prostatic region of the UGS was removed from the bladder at the bladder neck, and mesenchymal cells were isolated as described by Gupta (1999). Briefly, UGS tissue was disrupted by digestion with 3 mg collagenase type I/mL (Sigma Chemical Co., St. Louis, MO) for 30–50 min at 37°C in a shaking water bath followed by manual pipetting. Clumps of epithelium were allowed to settle out, and suspended mesenchymal cells were collected and cultured in complete medium [RPMI-1640 without phenol red (Gibco, Grand Island, NY) supplemented with 2 mM l-glutamine, 100 U penicillin G sodium/mL, 100 mg streptomycin sulfate/mL, and 0.25 mg fungizone/mL] with 10% (vol/vol) fetal bovine serum (FBS; U.S. Bio-Technologies, Parkerford, PA). Cells were grown to 95% confluence and then passaged by digestion with 0.05% trypsin in 0.53 mM EDTA (Gibco) for 5 min at room temperature. Cell viability was assayed with alamarBlue (BioSource International, Camarillo, CA) according to the manufacturer’s instructions.

We characterized the cell-type composition of the UGS cell primary cultures by immunofluorescent staining of cytokeratins with mouse anti-pan-cytokeratin clone PCK-26 fluorescein isothiocyanate conjugate (Sigma), and co-staining of the mesenchymal cell marker vimentin with goat anti-vimentin (Sigma) and rabbit anti-goat Cy3 conjugate (Sigma) (Prins et al. 1991).

During experimental treatments with E_2_, BPA, tamoxifen, and raloxifene, FBS was charcoal-stripped to remove all hormones, and cells were maintained in a constant background of 690 pM DHT (200 pg/mL). Cells were treated with DHT rather than testosterone to control for potential treatment effects on the intracellular concentration of this high-affinity ligand for the androgen receptor, which is formed from testosterone in UGS mesenchyme cells *in vivo*, and also to avoid the intracellular metabolism of testosterone to E_2_ by aromatase; DHT is not a substrate for aromatase (Kokontis and Liao 1999). First passage cells were seeded onto 24-well plates at 7 × 10^4^ cells/well in estrogen-free complete medium with 5% (vol/vol) charcoal-stripped FBS, 5% (vol/vol) charcoal-stripped horse serum (Sigma), 690 pM DHT (Steraloids, Wilton, NH), and ITS supplement (insulin-transferrin-selenium; Cambrex, Walkersville, MD) for final concentrations of 10 μg insulin/mL, 10 μg transferrin/mL, and 10 ng selenium/mL. Cells were maintained in this estrogen-free medium for 3 days, with one medium change, before the start of treatments. E_2_, BPA, and tamoxifen were obtained from Sigma. Raloxifene (LY 156,758) was obtained from Eli Lilly (Indianapolis, IN). During treatments with E_2_ and BPA, cells were grown for 4 days, and the medium was changed every day, except where noted. The concentration of E_2_ in culture medium during treatments was measured by radio-immunoassay as previously described by vom Saal et al. (1990).

### Real time RT-PCR measurement of gene expression

Total RNA was isolated with the RNAqueous kit (Ambion, Austin, TX) according to the manufacturer’s instructions. Total RNA was quantified by absorbance at 260 nm. Expression of specific mRNAs were measured by one-step real-time reverse transcription-polymerase chain reaction (RT-PCR) as described by Bustin (2000), with the TaqMan EZ RT-PCR kit (PE Applied Biosystems, Foster City, CA) on the ABI PRISM 7700 Sequence Detection System (PE Applied Biosystems). The concentrations of Mn^2+^, probe, and primers were optimized for each primer/probe set. Primer/probe sets for *Ar*, vimentin (*Vim*; GenBank accession no. NM_011701.3), and acidic ribosomal phosphoprotein P0 (*Arbp*; GenBank accession no. NM_007475.2) were designed using Primer Express software (PE Applied Biosystems) and are shown in Table 1. Primers were designed to span exon boundaries in order to prevent amplification of genomic DNA. Primers were synthesized by Invitrogen (Carlsbad, CA), and probes were synthesized by PE Applied Biosystems. The primer/probe set for *Esr1* was TaqMan Gene Expression Assay ID Mm00433149_m1 (PE Applied Biosystems), which spans *Esr1* exons 3–4.

The relative concentrations of specific mRNAs in each sample were normalized to total RNA per well, as described by Bustin (2000) and Latil et al. (2001). Normalization to total RNA allowed for comparisons between independent experiments. In parallel experiments, total DNA per well was measured by fluorescence of Hoechst 33258 (Sigma), as described by Labarca and Paigen (1980). From these data, the average RNA/DNA ratio was calculated for each treatment; we used these values to convert the mRNA/total RNA measurements to mRNA/DNA to assess the effect of E_2_ and BPA on gene expression per cell.

### Statistical analyses

Treatments were replicated in three wells per experiment and at least two, and in most cases more (up to 10), replicate experiments. Outliers were detected with Grubbs’ test (Grubbs 1969) and removed. Treatment effects were evaluated on untransformed data for RNA, and on reciprocals for DNA and gene expression, with the analysis of variance (ANOVA) general linear model procedure using SAS software (SAS Institute Inc., Cary, NC). We made planned comparisons of means for each treatment relative to controls using the least-squares means test only if the overall ANOVA showed significant treatment effects. To avoid inflation of error rates, we did not use multiple comparisons among all treatments. The criterion for statistical significance was *p* < 0.05.

## Results

### Characterization of UGS cells and nominal E_2_ concentration in primary culture

Consistent with previous reports (Gupta 1999), immunofluorescent staining for the mesenchymal cell marker vimentin revealed no epithelial cells in first passage cells treated for 5 days with 0.1 nM E_2_ or with no E_2_ (data not shown). The UGS primary cell cultures that we examined were thus homogenous populations of mesenchyme cells that retained mesenchymal differentiation markers throughout the incubation period.

After the initial administration of E_2_ in culture medium, the E_2_ concentration slowly decreased, presumably by metabolism, sequestration in cells, and/or binding to the tissueculture dish. In more detail, E_2_ was administered at a concentration of 1 nM, in the middle of the dose range in our experiment. The concentration of E_2_ in medium decreased by 2 hr to approximately 90% and by 18 hr to approximately 60% of the administered dose, and then remained stable through the remaining time period examined (up to 48 hr).

**Figure 1:**
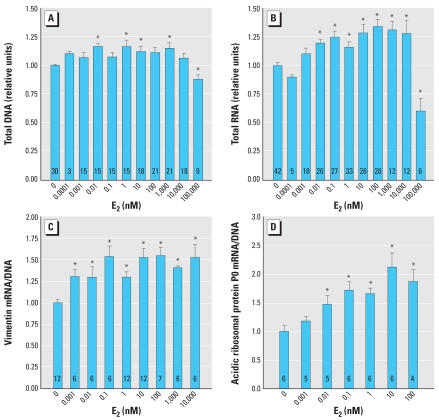
Indicators of cell growth increase with E_2_ dose. (*A*) DNA content slightly increased with E_2_ in a dose-independent manner up to 10,000 nM and decreased significantly at 100,000 nM E_2_. (*B*) Total RNA content increased with E_2_ in a dose-dependent manner up to 10,000 nM and decreased significantly at 100,000 nM E_2_. (*C* and *D*) Gene expression of the cytoskeleton protein vimentin (*C*) and the ribosomal component acidic ribosomal phosphoprotein P0 (*D*) increased with E_2_ treatment. Units are fold induction relative to the control. Error bars represent 1 SE. The number of replicates measured for each treatment is shown in each bar. *Values significantly different from the control (*p* < 0.05).

**Figure 2:**
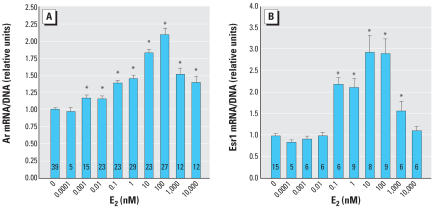
Biphasic *Ar* and *Esr1* gene expression responses induced by E_2_. (*A*) Ar mRNA expression increases with increasing dose of E_2_ up to a dose of 100 nM. A dose of 0.001 nM (0.27 pg/mL) is within the physiologic range of free E_2_ in mouse fetuses, and a significant response to this dose is consistent with prior *in vivo* findings (vom Saal et al. 1997). (*B*) Esr1 mRNA expression increases with increasing dose of E_2_ up to a dose of 100 nM. Error bars represent 1 SE. The number of replicates measured for each treatment is shown in each bar. *Values significantly different from the control (*p* < 0.05).

In the experiments we conducted, test chemicals were added to medium every 24 hr. Thus, at the midpoint of the dose–response curve tested, the actual E_2_ concentration in the culture medium was approximately 60% of the initial concentration at the time we collected the cells for analysis of mRNA. Measurement of DNA and RNA content and induction of gene expression confirmed that bioactive amounts of E_2_ were thus present at nominal concentrations < 0.001 nM (Figures 1 and 2).

### E_2_ induces growth of UGS mesenchyme cells

E_2_ treatment induced a small increase in cell growth and proliferation as indicated by DNA and RNA content at doses of 0.01–10,000 nM (Figure 1A, 1B). At 100,000 nM E_2_, inhibition of cell growth and proliferation was observed (Figure 1A, 1B). Cell viability was not affected at any E_2_ dose tested (data not shown). Subsequent experiments used a dose range of 0.0001–10,000 nM in order to avoid the cell growth–inhibiting effects of very high doses of E_2_. Relative total RNA was induced to a greater degree than DNA (Figure 1A, 1B). The housekeeping genes *Vim*, a component of the cytoskeleton in mesenchyme cells, and *Arbp*, a component of the ribosome, were examined to determine whether either could be used as a reference gene. However, both of these genes increased expression in response to E_2_, consistent with a general induction of cell growth (Figure 1C, 1D). Vimentin and acidic ribosomal phosphoprotein P0 exhibited differently shaped dose–response curves, suggesting differences in their mechanisms of transcriptional regulation by E_2_. Because neither house-keeping gene was an appropriate control gene, expression of specific mRNAs was normalized to DNA.

### E_2_ induces the steroid receptor mRNAs Ar and Esr1

Ar mRNA expression was induced by E_2_ up to just over 2-fold above control levels (Figure 2A). The observed threshold of induction, 0.001 nM, is slightly higher than the measured free serum E_2_ concentration of 0.00077 nM or 0.21 pg/mL in male mouse fetuses on GD18 (vom Saal et al. 1997). The increase in Ar mRNA with E_2_ dose was monotonic up to 100 nM E_2_. At doses of ≥ 1,000 nM E_2_, Ar mRNA levels declined relative to the maximum observed induction at 100 nM E_2_. Inhibition of cell growth was only evident at 100,000 nM E_2_.

**Figure 3:**
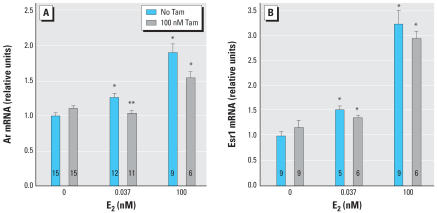
Antiestrogen treatment inhibits E_2_-induced expression of *Ar* but does not significantly inhibit E_2_-induced expression of *Esr1*. (*A*) The antiestrogen tamoxifen (Tam) blocks induction of Ar mRNA by a physiologic dose of E_2_, and the inhibition by Tam is overcome by a pharmacologic dose of E_2_. (*B*) Tam does not significantly inhibit induction of Esr1 mRNA by a physiologic dose of E_2_, or by a pharmacologic dose of E_2_. Error bars represent 1 SE. The number of replicates measured for each treatment is shown in each bar. *Values significantly different from the control (*p* < 0.05). **Significant differences between the same E_2_ treatment with and without Tam (*p* < 0.05).

The induction of Ar mRNA by a physiologically relevant level of E_2_, 0.037 nM (10 pg/mL), was significantly inhibited by antiestrogen treatment (Figure 3A). The anti-estrogen raloxifene (100 nM) had similar effects to 100 nM tamoxifen (raloxifene data not shown). The inhibition of the *Ar* response to E_2_ by tamoxifen was overcome by addition of a pharmacologic dose of 100 nM E_2_, demonstrating that the inhibition by tamoxifen observed at 0.037 nM E_2_ is not due to cytotoxicity or other nonspecific effects (Figure 3A).

Esr1 mRNA expression was induced by E_2_ by approximately 3-fold over the control, with a threshold at 0.037 nM (Figure 3B) and a peak at 10–100 nM E_2_ (Figure 2B). The induction of Esr1 mRNA by 0.037 nM E_2_ was not significantly inhibited by anti-estrogen treatment (Figure 3B).

### BPA acts as an estrogen agonist in UGS mesenchyme cells

**Figure 4:**
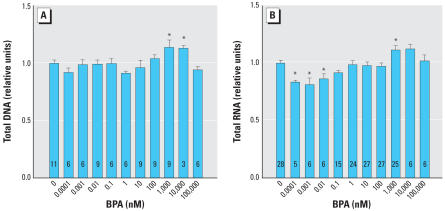
Indicators of cell growth in response to BPA. (*A*) Total DNA content was stable at low doses of BPA and significantly increased only at 1,000 nM. (*B*) Total RNA was significantly decreased at very low doses of BPA, 0.0001–0.001 nM, and significantly increased only at 1,000 nM. Error bars represent 1 SE. The number of replicates measured for each treatment is shown in each bar. *Values significantly different from the control (*p* < 0.05).

**Figure 5:**
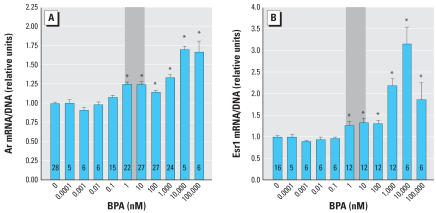
Gene expression of *Ar* and *Esr1* are induced by the synthetic estrogen BPA. (*A*) Ar mRNA expression increases with increasing dose of BPA. (*B*) Biphasic Esr1 mRNA expression response increases with increasing dose of BPA up to a dose of 10,000 nM (10 μM or 2.28 μg/mL). Shaded areas represent the typical range of concentrations of unconjugated BPA measured in human serum (Welshons et al. 2006). Error bars represent 1 SE. The number of replicates measured for each treatment is shown in each bar. *Values significantly different from the control (*p* < 0.05).

**Figure 6:**
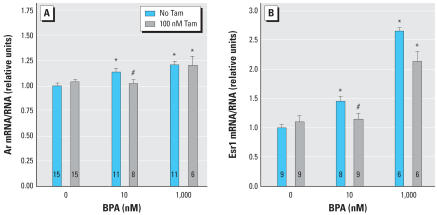
Antiestrogen treatment inhibits BPA-induced expression of steroid receptors *Ar* and *Esr1*. (*A*) The antiestrogen tamoxifen (Tam) blocks induction of Ar mRNA by a 10-fold lower dose of BPA, and the inhibition by tamoxifen is overcome by a 10-fold higher dose of BPA. (*B*) Tam inhibits induction of Esr1 mRNA by a 10-fold lower dose of BPA, and the inhibition by Tam is overcome by a 10-fold higher dose of BPA. Error bars represent 1 SE. The number of replicates measured for each treatment is shown in each bar. *Values significantly different from the control (*p* < 0.05). **Significant differences between the same BPA treatment with and without Tam (*p* < 0.05).

The effects of BPA on cell growth as indicated by DNA and RNA content (Figure 4) were much less pronounced than the effects of E_2_ (Figure 1). The dose–response curve for RNA was biphasic, with significant reductions in RNA content, but not DNA, at very low concentrations of BPA (Figure 4). Ar and Esr1 mRNA expression were induced by BPA treatment (Figure 5). The dose–response curves for BPA were shifted to the right by approximately 1,000-fold for Ar and approximately 30-fold for Esr1, relative to E_2_ (based on the significant stimulation of Esr1 in response to 0.037 nM E_2_; Figure 3B). As indicated in Figure 5A and 5B, a significant increase in both *Ar* and *Esr1* transcription occurred at BPA concentrations within the range typically reported in human blood and tissues, including fetal blood (Schönfelder et al. 2002; Welshons et al. 2006). The induction of both Ar and Esr1 mRNAs by a physiologically relevant low dose of BPA (10 nM) was inhibited by a 100-nM dose of tamoxifen. For both genes, the inhibition by tamoxifen was overcome by a high dose of BPA (1,000 nM) (Figure 6).

## Discussion

The aims of the present study were to investigate whether there were direct effects on *Ar* and *Esr1* gene activity that could be related to the previously observed stimulatory effect on prostate development caused by prenatal exposure to serum concentrations of bioactive E_2_ in fetal mice and the concentration of bioactive BPA currently measured in human fetal serum. We found that both the natural estrogen E_2_ and the synthetic estrogenic endocrine disruptor BPA stimulated increases in prostate Ar and Esr1 mRNAs. The stimulation occurred at physiologically relevant part-per-trillion doses of E_2_, and at parts-per-billion doses of BPA, which are within the range found in human fetal blood (reviewed by Welshons et al. 2006).

Exposure of male mouse and rat fetuses to slightly elevated estrogen levels results in permanent prostate enlargement and elevated androgen receptor levels in adulthood (Timms et al. 1999; vom Saal et al. 1997). Some confusion concerning effects of estrogen on the prostate has been created by studies in which only very high, pharmacologic (supraphysiologic) doses of estrogen were tested. Effects of pharmacologic doses of estrogenic chemicals on prostate development are not predictive of effects at physiologic doses. The dramatic effects of physiologic doses of estrogens have been revealed in studies in which pregnant mice were administered low doses of E_2_, the drugs diethylstilbestrol (DES) and ethinylestradiol, BPA, or the estrogenic insecticide methoxychlor, resulting in a permanent increase in prostate size in male offspring (Gupta 2000; Nagel et al. 1997; Thayer et al. 2001; vom Saal et al. 1997; Welshons et al. 1999). As the dose of E_2_ or DES was increased into the pharmacologic range, the stimulating effect observed at low doses disappeared and inhibition of prostate development occurred (Gupta 2000; Timms et al. 2005; vom Saal et al. 1997). Thus, the inhibitory effects of pharmacologic doses of estrogens on the developing prostate are opposite to effects of physiologic doses of the same estrogenic chemicals.

Available data on short-term effects of developmental estrogen exposures are consistent with the long-term effects observed in adulthood. For example, a high, pharmacologic dose of estradiol benzoate given to neonatal rats induced down-regulation of androgen receptor protein expression as early as postnatal day (PND) 6 (Prins and Birch 1995). In contrast, low, physiologically relevant doses of estrogenic chemicals (DES and BPA) fed to pregnant mice induced up-regulation of prostatic androgen receptor ligand binding activity in male offspring as early as PND3; this observation was replicated in organ culture of fetal mouse prostate treated with 0.1 pg/mL DES or 50 pg/mL BPA (Gupta 2000). The increase in prostate size in response to 50 pg/mL (0.22 nM) BPA in organ culture is below the threshold observed for stimulation of either *Ar* or *Esr1* gene expression observed in the present study.

Our findings support the hypothesis that prenatal exposure to elevated estrogen levels permanently increases prostate size and androgen responsiveness at least in part by inducing Ar mRNA expression. Importantly, the effects on Ar mRNA expression occurred with a threshold at 0.001 nM E_2_ (0.28 pg/mL), consistent with concentrations previously shown to alter prostate development *in vivo.* Specifically, the total serum E_2_ concentration in male mouse fetuses on GD18 is approximately 0.35 nM (94 pg/mL), and the free (unconjugated and unbound to plasma proteins) serum concentration of E_2_ is 0.00077 nM (0.21 pg/mL), or 0.2% of total serum E_2_ (vom Saal et al. 1997), similar to findings in rats (Montano et al. 1995). An increase in free serum E_2_ in male mouse fetuses (due to maternal administration of E_2_ via Silastic capsule) to 0.0012 nM (0.31 pg/mL) significantly increased prostate size and the number of prostatic androgen receptors in adulthood (vom Saal et al. 1997). Our results show that at these same physiologic doses, E_2_ acts directly on fetal UGS mesenchyme cells to increase Ar mRNA expression. This response was inhibited by the antiestrogens raloxifene (data not shown) and tamoxifen (Figure 3), suggesting that the induction of Ar mRNA by E_2_ is mediated through the classical genomic estrogen receptor pathway.

The differences in the shapes of the dose–response curves for *Ar* and *Esr1* suggest that the receptors are regulated by distinct mechanisms. Distinct dose–response relationships were also noted for vimentin, acidic ribosomal protein P0, total RNA content, and DNA content. These findings are consistent with data from microarray studies demonstrating considerable diversity in dose–response relationships of different estrogen-responsive genes; in particular, as one moves across the dose–response curve, entirely different sets of genes are activated or inhibited (Coser et al. 2003; Shioda et al. 2006). Induction of *Ar* and *Esr1* also displayed different responses to inhibition by anti-estrogens, in that *Esr1* induction was not significantly inhibited by antiestrogen co-treatment (Figure 3). The threshold for effects on *Esr1* expression was between 0.01 nM (2.3 ng/mL) and 0.037 nM (8.4 ng/mL) E_2_ (Figures 2B and 3B). This is above the normal range of free E_2_ in serum in male mouse fetuses. However, serum estradiol may underestimate estrogen levels in prostate tissue because cells in the developing prostate express aromatase, which metabolizes testosterone to E_2_ (Ellem and Risbridger 2006; Risbridger et al. 2003), and because estrogen receptor agonists, including xenoestrogens, exhibit additive effects in combination (Rajapakse et al. 2002). The induction of *Esr1* expression by E_2_ suggests that estrogen exposure may create a positive feedback loop in the UGS, such that exposure to estrogens increases sensitivity to future or continuing exposure.

Although the observed effects of physiologic concentrations of E_2_ on Ar mRNA expression are consistent with established *in vivo* effects, our pharmacologic dose range (e.g., 100 nM) *in vitro* observations are not consistent with established *in vivo* effects (Gupta 2000; Prins and Birch 1995; vom Saal et al. 1997), which predicted a decline in Ar mRNA expression relative to control levels at this high dose of E_2_ (Figure 2A). The *in vivo* regulation of androgen receptors by pharmacologic doses of estrogens may thus involve systemic and posttranscriptional effects that are not observable in terms of Ar mRNA levels in isolated mesenchyme cells. The involvement of posttranscriptional mechanisms is supported by the observation that developmental exposure to pharmacologic doses of estrogens permanently up-regulates androgen receptor degradation by the proteasome (Woodham et al. 2003).

The behavior of BPA in this system is consistent with the established activity of BPA as an estrogen receptor agonist (reviewed by vom Saal and Hughes 2005; vom Saal and Welshons 2006; Welshons et al. 2006), which was first reported in 1936 (Dodds and Lawson). The weak effects of BPA on cell growth, measured as DNA and RNA content, are consistent with previous reports that the relative potency of BPA is greater in stimulating estrogen receptor–dependent gene transcription than in stimulating growth of uterine tissue (Nagel et al. 2001). There are several interesting differences between the dose–response curves of *Ar* and *Esr1* in response to BPA compared with E_2_. Based on the thresholds of induction of gene expression, BPA is approximately 1,000-fold less potent than E_2_ for induction of *Ar*, but only about 30-fold less potent than E_2_ for induction of *Esr1* (based on a threshold of *Esr1* induction of 0.037 nM E_2_; Figure 3B). In addition, the shape of the dose–response curve for *Esr1* differs between E_2_ and BPA; induction of *Esr1* by BPA was inhibited by tamoxifen, whereas induction of *Esr1* by E_2_ was not significantly inhibited by tamoxifen. These differences between E_2_ and BPA, which are seen in the *Esr1* dose–response curves but not the *Ar* dose–response curves, underline the probability that distinct mechanisms are at work in the induction of *Ar* and *Esr1* by estrogens.

Of great importance, the doses of BPA required for induction of both *Ar* and *Esr1* are within the range of typical levels of BPA measured in human serum, which range from approximately 1 to 10 nM (Figure 5) (Schönfelder et al. 2002; Welshons et al. 2006). Because our experiments measured the response to BPA in the absence of other estrogens, they are likely to underestimate the induction of *Ar* and *Esr1* expression in response to the additive mixture of endogenous estrogens, BPA, and other xenoestrogens to which humans are continuously exposed in our modern world (Colborn et al. 1993). The consequences of developmental induction of *Ar* and *Esr1* for the adult phenotype of the prostate have not been directly examined, but exposure during fetal life to very low doses of BPA (2–50 μg/kg/day) permanently increases prostate size in mice (Gupta 2000; Nagel et al. 1997). Neonatal exposure to a 10 μg/kg/day dose of BPA results in precancerous lesions (prostate interepithelial neoplasia) in adult male rats, associated with epigenetic changes (Ho et al. 2006). The report of Ho et al. (2006) is consistent with the finding that estrogenic chemicals stimulate an abnormal rate of proliferation in basal epithelial cells in the primary ducts of the mouse fetal prostate (Timms et al. 2005). Basal cells are progenitor cells proposed to be involved in prostate cancer (Kirschenbaum et al. 2006). We are currently examining whether the permanent increase in prostate AR receptor protein in mice exposed during fetal life to low doses of estrogenic chemicals is caused by a permanent increase in expression of the *Ar* gene, and whether this is associated with a change in DNA methylation at the *Ar* gene.

## Conclusions

Ar mRNA in mesenchyme cells isolated from fetal mouse prostate is up-regulated by E_2_ within its physiologic range, and by BPA within the range detected in human fetal serum. Induction of Ar mRNA by E_2_ or BPA was inhibited by antiestrogen co-treatment. Therefore, the low-dose effects of estrogens on prostatic *Ar* regulation are estrogen receptor–dependent, act at the transcriptional level, are mediated through local effects on UGS mesenchyme cells, and can be modeled in a primary cell culture system. In contrast, down-regulation of androgen receptor protein in response to high doses of estrogens *in vivo* likely includes systemic and post-transcriptional mechanisms. Esr1 mRNA is also up-regulated by E_2_ and BPA in a dose-dependent manner, suggesting the possibility of positive feedback in estrogen effects on the prostate. The induction of *Esr1* by E_2_ is not significantly inhibited by antiestrogen treatment, suggesting the involvement of non-estrogen receptor–mediated mechanisms.

Taken together, these results are consistent with the hypothesis that prenatal exposure to elevated estrogen or xenoestrogen levels within the physiologic range results in an increase in androgen receptor and estrogen receptor 1 (α ) number in the developing prostate mesenchyme, which increases androgen and estrogen responsiveness and growth. The estrogen receptor–dependent induction of *Ar* by BPA confirms that this mechanism is not unique to E_2_ and underscores the vulnerability of the developing reproductive system to the additive effects of exogenous estrogenic endocrine disruptors.

## Figures and Tables

**Table 1:** Sequences of primers and probes for real time RT-PCR assays.

Gene	Sequence (5′ → 3′)	5′ position in CDS	Exon boundary
*Ar*			
Forward	TGTCAAAAGTGAAATGGGACC	1494	
Reverse	TGGTACTGTCCAAACGCATGT	1567	1–2 at 1553
Probe	TGGATGGAGAACTACTCCGGACCTTATGGG	1516	
*Arbp*			
Forward	GAGATTCGGGATATGCTGTTGG	289	2–3 at 302
Reverse	GGCGATGGCACCAGCC	351	
Probe	CAATAAGGTGCCAGCTGCTGCTCG	312	
*Vim*			
Forward	GCACCCTGCAGTCATTCAGA	602	
Reverse	CCACTTTCCGTTCAAGGTCAA	673	3–4 at 660
Probe	AGGATGTTGACAATGCTTCTCTGGCACG	623	

CDS, coding sequence.
